# Development and field application of metabarcoding-adapted mt-ND4 markers shows substantial gene flow and varying local pressures on *Haemonchus contortus* and *Teladorsagia circumcincta* populations in the UK

**DOI:** 10.1371/journal.pone.0327254

**Published:** 2025-07-02

**Authors:** Osama Zahid, Umer Chaudhry, Neil Donald Sargison

**Affiliations:** 1 University of Edinburgh, Royal (Dick) School of Veterinary Studies and Roslin Institute, Easter Bush Veterinary Centre, Midlothian, United Kingdom; 2 University of Calgary, Faculty of Veterinary Medicine, Foothills Campus, Hospital Drive NW, Calgary, Canada; 3 College of Veterinary Medicine, Long Island University, New York United States of America; Beni Suef University Faculty of Veterinary Medicine, EGYPT

## Abstract

Gastrointestinal nematodes impose a significant burden on livestock production and public health by reducing animal productivity and increasing the environmental impact of farming. Modern sequencing techniques enable the efficient exploration of genetic diversity, necessary to inform effective parasite control. In this study, we developed and validated new mitochondrial ND4-based markers adapted for high-throughput sequencing. This enabled detailed analysis of genetic diversity in two important nematode species, *Haemonchus contortus* and *Teladorsagia circumcincta*. Laboratory validations confirmed that the assay reliably detected as little as 1% of larvae in mixed samples and accurately identified strain variants. Field application on 30 sheep farms across England and Scotland revealed 60 unique genetic variants in *H. contortus* and 35 in *T. circumcincta*. A single variant dominated the sequence reads in both species, particularly *T. circumcincta*. Regional comparisons showed that *H. contortus* exhibited fewer yet persistent variants in Scotland than in England; while phylogenetic analyses indicated a common origin and significant gene flow between regions. In contrast, *T. circumcincta*, despite being more prevalent across all farms, displayed lower overall diversity with a shared dominant variant; evidence of dual origins and marked regional differences in evolutionary pressures. Comparisons with publicly available global sequence data revealed distinct clustering of *H. contortus* isolates, separating Asian sequences from those in the United Kingdom and Australia. *T. circumcincta* isolates showed no apparent geographic clustering. These findings demonstrate the potential of high-throughput mitochondrial marker analysis to unravel complex parasite population dynamics, and to inform sustainable management strategies in the face of challenges such as drug resistance and climate change.

## Introduction

Gastrointestinal nematodes (GINs) are parasitic roundworms that significantly impact livestock and human health, making them a critical concern in the global livestock industry and the ‘One Health’ initiative [1]. These parasites negatively impact animal health and productivity by causing weight loss, anaemia and weakness; leading to poor feed conversion, decreased weight gains, and reduced product quality [1,2]. Their presence also exacerbates the carbon footprint of the industry [3,4].

There are considerable challenges involved in understanding GIN infections. The co-existence of multiple GIN species in diverse parasitic communities, each with unique epidemiological and pathogenic profiles [5–9], along with environmental factors and farming practices, significantly influence infection dynamics. Climate change adds another layer of complexity by altering epidemiological patterns and potentially undermining established management strategies [10–12]. The growing issue of anthelmintic resistance among various GIN species further complicates this scenario [13–15], making it increasingly crucial to monitor dynamic infection patterns and genetic diversity within and across GIN communities.

Advances in high-throughput sequencing technologies have revolutionised our ability to study GIN population dynamics and genetic diversity. Coupled with the development of robust genetic markers and metabarcoding techniques, these innovations enable the simultaneous and cost-effective analysis of hundreds of GIN populations. One such example is the ‘nemabiome’ approach, which uses Illumina metabarcoding to analyse the rDNA ITS-2 region. This has proven highly effective for accurate molecular species identification of all clade V nematode species within different populations [16], allowing researchers to evaluate the effects of various management and environmental factors on each species.

While the ‘nemabiome’ offers a fast and reliable method for species identification, it represents only the initial step toward a deeper understanding of genetic diversity; a critical factor for developing effective and sustainable strategies to control GIN infections amid evolving challenges. Mitochondrial DNA (mtDNA) markers, such as those based on cytochrome oxidase I (COI) and NADH dehydrogenase (ND) genes, can be employed to take it a step further. Mitochondrial DNA is highly diverse because of its high mutation rate [17,18], yet easy to trace because of a lack of recombination [19]; making these markers ideal for monitoring the genetic diversity and infection patterns of different GIN species. Hence, they have long been employed to explore genetic diversity, trace evolutionary histories, and examine population structures in GINs [20–24]. However, a gap remains in adapting these markers to modern high-throughput sequencing techniques for a much broader and cost-effective application.

To bridge this gap, the present study describes the development and validation of Illumina metabarcoding-adapted mt-ND4 primers for two economically important GIN species affecting small ruminants: *Haemonchus contortus* and *Teladorsagia circumcincta*. Additionally, the use of a primer multiplexing strategy [25,26] was explored, which allows for the simultaneous processing of samples from both species in a single assay. This approach has the potential to improve the efficiency of genetic studies on GIN populations by reducing the complexity and cost of sample processing, thus enhancing the scalability of research.

In practical application, these primers were used on field samples collected from 30 Scottish and English sheep farms to explore genetic diversity and infection patterns across regions. *T. circumcincta* is the most common GIN species in both regions, with reports of multidrug resistance (MDR) [27]; while *H. contortus* is uncommon in Scotland but more prevalent in England, with a comparatively unknown resistance level. This contrast provided a valuable opportunity to consider the behavioural patterns of these parasites under different conditions. The results were also compared with previously available sequence data from other countries. Overall, this comprehensive approach aimed to advance the scientific understanding of the techniques and demonstrates potential applications in developing a better understanding of GIN population dynamics.

## Materials and methods

S1 Fig shows a flowchart of the steps involved in the development and validation of primers, discussed in detail below.

### Parasite material and DNA extraction

For testing and validation of primers, sixteen laboratory strains were used: nine strains of *H. contortus* and seven of *T. circumcincta*. The *H. contortus* strains were MHco3 (ISE), MHco4 (WRS), MHco5 (IRE), MHco10 (CAVR), MHco16, MHco17, MHco18 (UGA2004), MFie13a, and MFie17. The *T. circumcincta* strains were MTci2 (CVL), MTci5, MTci7, MTci11, MTci12, MTci13, and MTci18. Additionally, an undocumented lab sample of *T. circumcincta* with an unknown strain identity was included. MHco3 and MTci2 were used for initial primer and multiplex development, while all strains were used for analysing and confirming the results in the next step. After their acquisition, the third-stage larvae (L_3_) of these strains were transported to the University of Edinburgh, Royal (Dick) School of Veterinary Studies (R(D)SVS). The L_3_ were either used immediately for DNA extraction, or preserved at −80°C for future use.

Additionally, field samples from 30 sheep farms across England and Scotland were collected during the summers of 2021 and 2022. Around 30 animals were sampled from each farm. Faecal samples were collected, stored in small plastic bags for individual animals, and sent to the R(D)SVS. Samples were processed by faecal egg counts (FEC) and DNA extraction within 24 hours of receipt. FECs were performed using a modified salt flotation and cuvette method [28], with a detection threshold of three eggs per gram (epg) of faeces. The eggs were extracted and washed in tap water before being incubated in tap water at room temperature for 36–48 hours to allow hatching into first-stage larvae (L_1_). Larval isolation was facilitated by a mini-Baermann apparatus setup. L_1_ were either immediately processed for DNA extraction, or stored at −80°C until further use.

Approximately 200 L_3_ were used for DNA extraction from laboratory isolates, and three replicates of each strain were prepared. The undocumented *T. circumcincta* sample was incorporated to test if the multiplex could effectively identify its origins. For field samples, the number of L_1_ used varied depending on the FEC and number of animals sampled, generally 10–15 times the mean FEC.

A worm lysis solution was prepared by mixing 1000 μl of Direct PCR Lysis Reagent (Viagen), 50 μl of proteinase K (Qiagen) solution, and 50 μl of 1M dithiothreitol (DDT). Each sample was placed in 20 μl of this lysis solution and was incubated at 60°C for two hours, followed by 15 minutes at 85°C to inactivate the proteinase K [29]. The resulting lysates were stored at −20°C or −80°C, depending on the time until further analysis.

An rDNA ITS-2 ‘nemabiome’ analysis was performed on field samples, following standard protocols [30], to identify and quantify the GIN species present. This analysis identified seven GIN species, including both *H. contortus* and *T. circumcincta*. *T. circumcincta* was found on all 19 English and 11 Scottish farms. In comparison, *H. contortus* was prevalent in English farms but missing on the majority of the Scottish farms. The results from individual farms are presented in S2 Fig.

### Reference library development and primer identification

NADH dehydrogenase subunit 4 (ND4) primers were developed for *H. contortus* and *T. circumcincta* (S1 and S2 Tables)*.* This specific subunit was picked because of its diverse nature and the availability of required reference sequences on the NCBI Genbank. Initially, the reference library contained 16 sequences for *H. contortus* and 23 for *T. circumcincta*, which were aligned using Geneious Prime software [31]. From these alignments, three sets of potential forward and reverse primers were manually identified for each species.

The initial reference library was updated as new mt-ND4 sequences became available on NCBI GenBank, enriching the database and enhancing the robustness of the primers’ validation process. The final library, detailed in S1 and S2 Tables, includes these additional references and was used to compare and validate the sequences obtained during the study.

### Primer testing and multiplexing

The development and testing of mt-ND4 primers for *H. contortus* and *T. circumcincta* involved several steps. Initially, various combinations of forward and reverse primers were tested through gradient PCR and gel electrophoresis to identify effective pairs. Once successful combinations were identified, the PCR products were verified through Sanger sequencing to ensure the correct genomic regions were amplified. The final selections included an *H. contortus* primer set producing a 247 base pair product and a *T. circumcincta* primer set with a 385 base pair product.

An adapter sequencing tag was added to each primer to prepare for sequencing on the Illumina platform. Four variants (0N, 1N, 2N, 3N) were created for each primer by appending extra random nucleotides between the locus-specific primer sequence and the Illumina adapter sequence, as shown in S2 Table. This modification was intended to increase the diversity of the generated amplicons and prevent sequencing channel oversaturation.

Subsequently, equal quantities of different variants (0N, 1N, 2N and 3N) of each primer were mixed and gradient PCRs were employed to determine the optimal annealing temperatures for the adapter PCR, testing temperatures of 55°C, 60°C, and 65°C. Both the primer sets worked at 55°C and 60°C. To integrate these primer sets into a multiplex, an equal mix of primers for both species was tested using gradient PCR, pinpointing 57°C as the optimal annealing temperature for the multiplex setup.

To evaluate the sensitivity of the multiplex to different DNA concentrations, L_3_ of *H. contortus* and *T. circumcincta* were mixed in various proportions (0:100, 25:75, 50:50, 100:0). Gel electrophoresis showed that the *T. circumcincta* primers were more efficient at amplifying DNA, which led to adjustments in the mixing ratios while maintaining a total primer concentration at 20µM. A 30:70 ratio of *T. circumcincta* to *H. contortus* was selected for subsequent experiments, based on the gel electrophoresis results.

Subsequently, a broader range of known larvae mixtures was analysed to assess the multiplex’s ability to detect varying DNA quantities from low numbers or ratios of larvae of a species within a sample. This phase involved creating 12 triplicate samples with diverse proportions of the two nematode species each containing a total of 200 L_3_ (excluding the negative controls) as depicted in S3 Fig alongside the gel electrophoresis results of their PCR products. The samples (1–12) contained 0:200 2:198, 10:190, 20:180, 50:150, 100:100, 15:50, 180:20, 190:10, 198:2, 200:0 and 0:0 larvae for *H. contortus*: *T. circumcincta*, respectively.

The conditions for adapter PCR followed standard metabarcoding protocols [32]. The PCR products underwent purification using Agencourt AMPure XP Magnetic Beads at a 1X ratio (Beckman Coulter, Inc.), employing a DynaMag magnetic stand, followed by a limited-cycle PCR to append unique “barcodes” or indices and P5/P7 sequencing tags to each adapter, facilitating sample identification during Illumina sequencing [33].

PCR products from each sample were then pooled in equal quality (10μl each) and subjected to extraction from 1.5% agarose gel and purification using a Qiagen kit, followed by a secondary purification with AMPure XP magnetic beads. The collective 50 μl of this pooled sample was submitted to Edinburgh Genomics, where it was quantified using the KAPA qPCR library quantification kit and sequenced on an Illumina Mi-Seq Sequencer with a 500-cycle pair-end reagent kit. The sequencing concentration was set at 15 nM, supplemented by an additional 15% PhiX Control v3 to ensure sequence diversity and accuracy.

The application of mt-ND4 multiplex to field samples presented unexpected challenges as the results from gel electrophoresis were inconsistent, showing complete failure in some instances and partial success in others. After extensive troubleshooting, it was discovered that the multiplex worked to some degree when applied to a low number of samples (<16), but failed in larger batches, indicating some non-specific interactions between primer sets and/or the template DNA making the process time-sensitive.

The samples were also processed using individual *H. contortus* and *T. circumcincta* primers alongside the multiplex to ensure high-quality data presentation. This improved the results for *T. circumcincta* significantly, while *H. contortus* results were comparable to those achieved through multiplexing. Regardless, in the interest of quality and consistency, the data presented in the results section are based on analyses conducted with individual primers.

### Post-sequencing analysis

For the post-sequencing analysis of mitochondrial amplicon data from *H. contortus* and *T. circumcincta*, the Mothur software suite [34] was used to run the previously published processing pipeline [35]. This pipeline efficiently joins paired forward and reverse sequence reads from Illumina sequencing, removes sequences with ambiguous bases, and excludes reads that are either too long or too short. After screening, sequences were aligned against the reference library, and identical sequences were grouped into Amplicon Sequence Variants (ASVs). This process culminated in the creation of a FASTA file containing all identified ASVs and a count table detailing the read counts for each ASV across the processed samples.

To streamline the analysis and ensure clarity, a cutoff point of 0.5% of the total reads was established for ASV inclusion for field samples, mitigating noise and the risk of errors such as ‘bleeding effects’ [36]. Additionally, to ascertain the identities of ASVs, an NCBI BLAST analysis was employed in addition to the Mothur analysis, offering a rigorous confirmation process.

The data were further organised and analysed using R software (https://www.R-project.org/), where the FASTA file and count table were integrated. ASVs were named based on their descending order of relative abundance, and read numbers were calculated for different farms and locations. This integration facilitated the generation of abundance charts of ASV distribution across various categories. Additionally, bootstrap resampling was performed to confirm the stability of these findings, ensuring that the observed differences are robust despite the disparity in sample size, with 11 Scottish farms compared to 19 English farms.

An automated R-loop [37] was used to generate group-specific FASTA files, which were then used in DNA polymorphism analysis and neutrality tests through DnaSP6 [38]. Genetic diversity metrics (number of sequences, ASVs, average sequences per ASV, ASV diversity, mutations, nucleotide diversity, and neutrality tests) were statistically compared between locations. Data normality was assessed, and t-tests or Mann-Whitney U tests were applied accordingly.

Neutrality tests, including Tajima’s D [39] and Fu and Li’s D* and F* [40], were used to evaluate evolutionary forces acting on populations. Positive neutrality test values indicated balancing selection or population contraction, while negative values suggested population expansion or purifying selection. To ensure computational feasibility, read counts for each ASV were scaled down by dividing by 500 and rounding to the nearest whole number, maintaining proportional ratios across ASVs and samples. While this adjustment removed some less common ASVs, its overall impact on results was minimal due to high sequence counts per ASV. Consistently applying this approach across all farms ensured equitable and informative comparisons.

In the final step, phylogenetic trees comprising all the ASVs were constructed using Geneious Prime [31], and these trees were further enhanced and annotated using the Interactive Tree Of Life (iTOL) [41], applying logarithmic transformation to the data to improve readability for tree annotations. In addition, another set of trees was produced to compare the ASVs found in the current study with the ones already available on NCBI Genbank (the reference library). These sequences were coloured based on the country of origin, and any identical sequences from the same country were merged.

## Results

### Validation of the multiplex assay with laboratory strains

The initial validation of the mt-ND4 multiplex assay, as depicted in S4 Fig, effectively demonstrates the assay’s ability to detect and quantify as low as 1% (2 out of 200) larvae of a single species in laboratory samples, with the mean read profile of triplicates closely matching the expected distributions. The negative control showed no sequencing reads across all replicates, confirming the assay’s specificity. S5 Fig illustrates the distribution of reads attributed to different ASVs through stacked area charts, which display consistency in the proportions of individual ASVs across different samples regardless of the total read count.

When applied to 9 *H. contortus* and 7 *T. circumcincta* laboratory strains, the data illustrated in S6 Fig shows consistent ASV proportions across replicates while highlighting distinct differences between the samples and strains. Remarkably, the analysis of the *T. circumcincta* sample with an unrecorded strain identity unequivocally matched it with the MTCI_02 strain. This underscores the method’s precision and reliability in distinguishing and accurately identifying different strains of GIN species.

### Field application

In the field application of the mt-ND4 on 11 Scottish and 19 English farms, a total of 60 unique *H. contortus* ASVs (named All_HC_1 through All_HC_60) and 35 unique *T. circumcincta* ASVs (named All_TC_1 through All_TC_35) were identified, after applying the filtration process outlined in the methods section.

**Fig 1:**
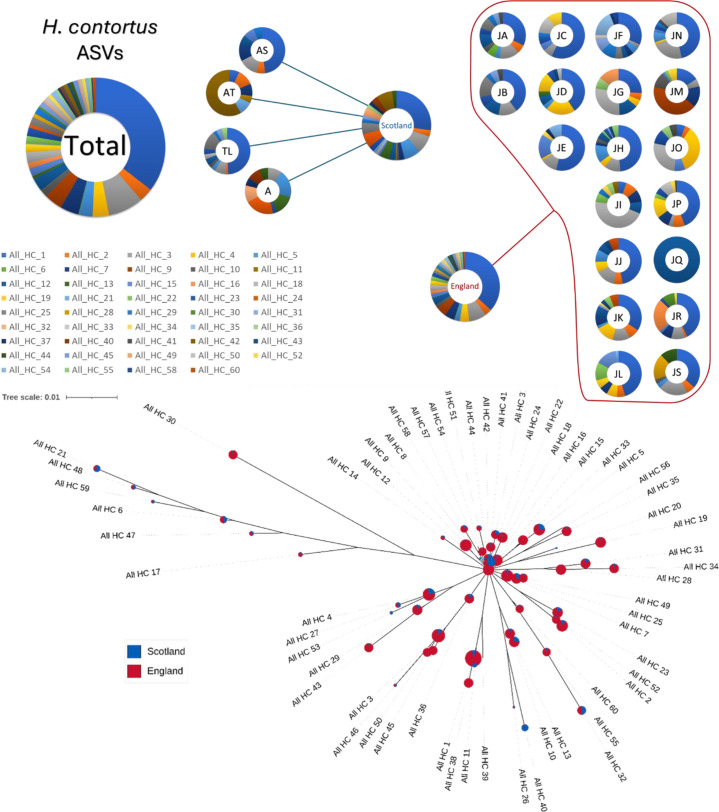
H. contortus mt-ND4 ASVs from Scottish and English samples. The top section of the figure displays pie charts representing individual farms, with each colour denoting one of 60 different mt-ND4 ASVs, and includes pie charts for Scottish, English, and all farms combined. Below, the phylogenetic tree illustrates the relationships among these ASVs. Each node, sized logarithmically based on read counts, represents a unique ASV, while the branches indicate the genetic distances between them.

*Haemonchus contortus* was detected on all 19 farms in England and four in Scotland, as shown in Fig 1. Notably, one Scottish farm (Farm AS) displayed detectable levels of *H. contortus* through mitochondrial analysis despite showing no signs of the parasite in the ITS-2 analysis (S2 Fig), underscoring the increased sensitivity of the mt-ND4 markers in detecting low-abundance species that might be overshadowed by more dominant species in rDNA ITS-2 assays. A single *H. contortus* ASV, All_HC_1, dominated, comprising over one-third of the total reads; and together with the next three most abundant ASVs, for more than half of all reads.

**Fig 2:**
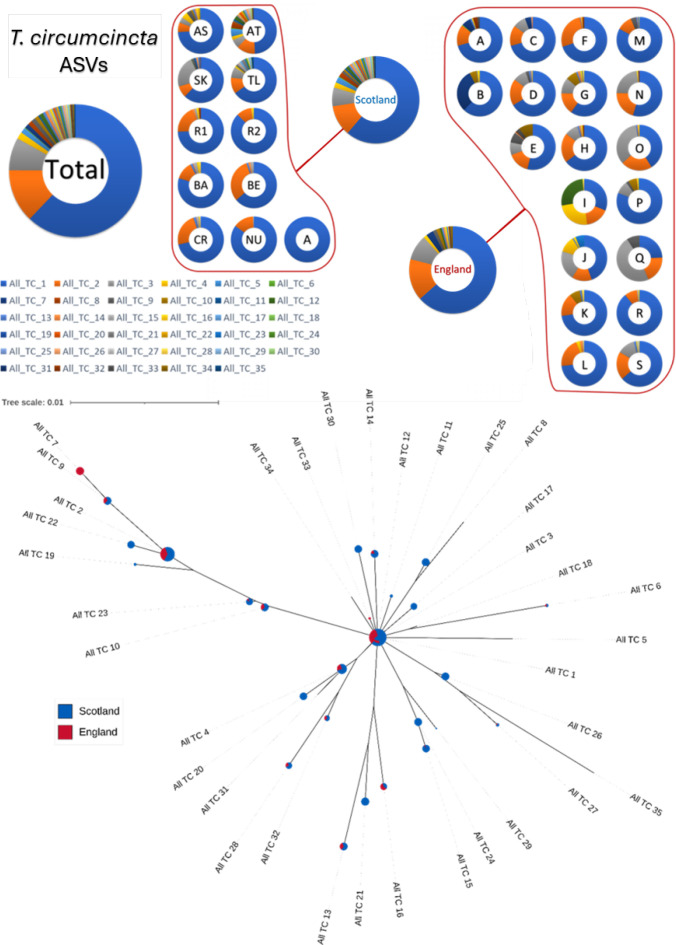
T. circumcincta mt-ND4 ASVs from Scottish and English samples. The top section of the figure displays pie charts representing individual farms, with each colour denoting one of 35 different mt-ND4 ASVs, and includes pie charts for Scottish, English, and all farms combined. Below, the phylogenetic tree illustrates the relationships among these ASVs. Each node, sized logarithmically based on read counts, represents a unique ASV, while the branches indicate the genetic distances between them.

For *T. circumcincta*, the results revealed even greater dominance by a single ASV, All_TC_1, which accounted for about 62% of the total sequence reads. The three most prevalent ASVs represented over 85% of the reads, indicating a significant concentration of genetic diversity within a few dominant variants. As shown in Fig 2, *T. circumcincta* was found on all 30 farms, which is also consistent with the rDNA ITS-2 analysis (S2 Fig).

### Regional distribution and diversity of *Haemonchus contortus*

The comparative analysis between Scottish and English farms reveals substantial differences in genetic diversity: Only 25 of the 60 *H. contortus* ASVs were identified in Scottish farms, complementing the lower prevalence compared to England. Most English farms, clustered within a 50 km radius of Oxford, predominantly featured a single ASV, All_HC_1, suggesting this variant’s long-term survival and prevalence. However, some English farms do show unique genetic profiles. For instance, farms G, I, M, O, and Q are dominated by All_HC_3, All_HC_9, All_HC_4, and All_HC_7, respectively. Notably, Farm Q has only a single ASV with a low read count of about 1,100, hinting at suboptimal DNA quality.

In contrast, Scottish farms present a broader array of ASV profiles, which could be attributed to the significantly different locations and possibly varying local environmental conditions. Farms AS (west coast) and TL (Isle of Mull) have compositions similar to English farms, suggesting some consistency in the genetic makeup across the regions. Conversely, Farm AT (Perthshire highlands) is notably distinct, primarily dominated by All_HC_42 and displaying only five other ASVs, reflecting a less diverse genetic structure. Farm A, operating as a small zoo in southwest Scotland, offers a unique perspective with a balanced diversity, where six different major ASVs each contribute roughly evenly to the total reads, indicating the effects of unique ecological or management influences.

Phylogenetic analysis shows a common origin for *H. contortus* across all farms (Fig 1). Scottish ASVs are either identical to, or closely derived from those found in English samples. This suggests limited genetic variation and historical gene flow between the areas, likely facilitated by frequent animal movements across the UK [42]. Such inter-regional gene flow appears to be significant, potentially even exceeding intra-regional gene flow despite the overall lower prevalence of the parasite in Scotland.

Table 1 presents metrics such as the number of sequences and ASVs, average sequences per ASV, ASV diversity, the number of mutations, and nucleotide diversity. A series of t-tests comparing these metrics between the regions revealed no statistically significant differences, with p-values consistently exceeding 0.05. The results remained unchanged after recalibrating the analysis to exclude Farm Q as a potential outlier.

**Table 1:** Genetic diversity metrics and neutrality tests of *H. contortus* populations in Scottish and English farms. This table presents the genetic diversity metrics and neutrality test results for *H. contortus* populations from various farms in Scotland and England, highlighting key differences between the two regions. The genetic diversity metrics include the number of sequences, number of ASVs, sequences per ASV, ASV diversity (Hd), the number of segregation sites (S), the number of mutations (Eta), and nucleotide diversity (π). The neutrality tests include Tajima’s D, Fu and Li’s D*, and Fu and Li’s F* statistics, along with their respective significance levels (SigD, SigD*, and SigF*). Statistical significance is denoted by ‘#’ for P < 0.10, ‘*’ for P < 0.05, ‘**’ for P < 0.01, and ‘***’ for P < 0.001. Additionally, ‘n.s.’ indicates results not statistically significant, and ‘n.d.’ denotes data not determined.

Farm	No. of sequences	No. of ASVs	Sequences per ASV	ASV diversity (Hd)	No. of Segregation sites (S)	No. of Mutations (Eta)	Nucleotide diversity (π)	TajimaD	SigD	FuLiD*	SigD*	FuLiF*	SigF*
**A**	27650	9	3072	0.861061	20	20	0.023727	2.486255	*	1.703089	*	2.435466	**
**AS**	10400	7	1486	0.726589	13	13	0.014436	1.53135	n.s.	1.47924	#	1.75678	*
**AT**	10950	6	1825	0.628964	20	20	0.017962	0.85659	n.s.	1.745407	**	1.638605	#
**TL**	34650	12	2888	0.724637	18	18	0.015638	1.238573	n.s.	1.612234	*	1.763745	*
**Scottish Mean**	**20913**	**8.5**	**2318**	**0.735313**	**18**	**18**	**0.017941**	**1.53**		**1.63**		**1.90**	
**A**	20800	12	1733	0.844219	25	25	0.020875	0.938633	n.s.	1.820078	**	1.738383	*
**B**	17150	8	2144	0.776908	16	16	0.017234	1.723087	n.s.	1.57977	*	1.94531	*
**C**	13250	7	1893	0.640709	15	15	0.013655	0.94888	n.s.	1.5534	*	1.558921	#
**D**	19550	9	2172	0.763053	20	20	0.019582	1.463497	n.s.	1.723559	**	1.930884	*
**E**	42300	7	6043	0.654872	14	14	0.013249	1.557075	n.s.	1.426521	#	1.790484	*
**F**	48500	12	4042	0.835596	18	19	0.019299	2.013045	#	1.546467	*	2.241474	**
**G**	15100	8	1888	0.808827	12	12	0.012104	1.316398	n.s.	1.407084	#	1.624323	#
**H**	36700	12	3058	0.724692	21	21	0.01654	0.952396	n.s.	1.719542	**	1.693142	#
**I**	15300	10	1530	0.759584	16	16	0.011952	0.400236	n.s.	1.586556	*	1.33056	n.s.
**J**	13500	8	1688	0.736059	17	17	0.015884	1.072995	n.s.	1.632598	*	1.670469	#
**K**	57100	13	4392	0.82998	21	21	0.017861	1.398929	n.s.	1.686596	*	1.909478	*
**L**	51050	8	6381	0.740783	16	16	0.017171	2.201647	*	1.500648	#	2.168874	**
**M**	66950	9	7439	0.758604	16	16	0.012713	1.147721	n.d.	1.479189	#	1.644231	#
**N**	31800	9	3533	0.711113	15	15	0.017398	2.311609	*	1.493885	#	2.190378	**
**O**	16350	8	2044	0.787546	14	14	0.013091	1.121873	n.s.	1.496805	#	1.604767	#
**P**	47350	10	4735	0.755974	19	19	0.019694	2.090945	#	1.627324	*	2.210111	**
**Q**	1100	1	1100	0	0	0	0	n.a.	n.a.	n.a.	n.a.	n.a.	n.a.
**R**	37550	9	4172	0.738702	20	21	0.017848	1.227481	n.s.	1.644824	**	1.801532	*
**S**	34300	7	4900	0.772195	15	15	0.016848	2.200254	*	1.488117	#	2.136823	**
**English Mean**	**30826**	**8.8**	**3415**	**0.717864**	**16.3**	**16.4**	**0.015421**	**1.45**		**1.58**		**1.84**	

The results of Tajima’s D test and Fu and Li’s tests (D* and F*) are also presented in Table 1. These metrics test for neutrality in the DNA sequences, where deviations from zero indicate different evolutionary forces acting on the population. The results generally show a positive trend, hinting at population contraction or balancing selection. For Scottish farms, Tajima’s D values ranged from 0.85 to 2.49, with Farm A displaying a significantly positive value (2.49, p < 0.05). For English farms, the values ranged from 1.41 to 2.31, with Farms L (2.20, p < 0.05), N (2.31, p < 0.05), and S (2.20, p < 0.05) showing significantly positive values. The Fu and Li tests (D* and F*) complement these results, showing even more significant differences due to their greater sensitivity to recent population changes.

### Regional distribution and diversity of *Teladorsagia circumcincta*

The comparative analysis of *T. circumcincta* between Scottish and English farms reveals minimal differences and a high gene flow with all major ASVs shared between the regions (Fig 2). Most farms in both Scotland and England typically show a predominance of All_TC_1, followed by All_TC_2. However, there are some exceptions. Farms J, O, and Q in England display a notably higher prevalence of All_TC_3, while Farm I features nearly equal prevalences across four different ASVs. Notably, Farm I also had the lowest mean FEC of all the English farms. In Scotland, Farm A stands out as it consists exclusively of one ASV, All_TC_1. This is also the only Scottish farm with a dominant prevalence of *H. contortus* (S2 Fig) and has multiple co-grazing hosts; explored in detail in a related study [43].

The phylogenetic analysis illustrates that the two major *T. circumcincta* ASVs, All_TC_1 and All_TC_2, are positioned on opposite branches of the tree, potentially suggesting two distinct origins of *T. circumcincta* in the region (Fig 2). The species exhibits greater diversity in Scotland, potentially due to the absence of competition from *H. contortus* and a better adaptation to the local environment.

Genetic diversity metrics and neutrality tests of *T. circumcincta* populations are shown in Table 2. The mean values for these metrics reveal notable differences between the regions. However, t-tests revealed only the differences between the number of sequences (t-statistic = 4.70, p-value < 0.001) and ASVs (t-statistic = 3.53, p-value = 0.0015) to be statistically significant.

**Table 2:** Genetic diversity metrics and neutrality tests of *T. circumcincta* populations from Scottish and English farms. This table presents the genetic diversity metrics and neutrality test results for *T. circumcincta* populations from various farms in Scotland and England, highlighting key differences between the two regions. The genetic diversity metrics include the number of sequences, number of ASVs, sequences per ASV, ASV diversity (Hd), the number of segregation sites (S), the number of mutations (Eta), and nucleotide diversity (π). The neutrality tests include Tajima’s D, Fu and Li’s D*, and Fu and Li’s F* statistics, along with their respective significance levels (SigD, SigD*, and SigF*). Statistical significance is denoted by ‘#’ for P < 0.10, ‘*’ for P < 0.05, ‘**’ for P < 0.01, and ‘***’ for P < 0.001. Additionally, ‘n.s.’ indicates results not statistically significant, and ‘n.d.’ denotes not determined.

Farm	No. of sequences	No. of ASVs	Sequences per ASV	ASV diversity (Hd)	No. of Segregation sites (S)	No. of Mutations (Eta)	Nucleotide diversity (π)	TajimaD	SigD	FuLiD*	SigD*	FuLiF*	SigF*
**A**	5400	1	5400	0	0	0	0	n.a.	n.a.	n.a.	n.a.	n.a.	n.a.
**AS**	5000	4	1250	0.52	6	6	0.004034632	−0.06714	n.s.	1.229063	n.s.	0.912833	n.s.
**AT**	8000	10	800	0.697436	13	13	0.005298035	−1.03773	n.s.	−1.37685	n.s.	−1.3968	n.s.
**BA**	10600	6	1767	0.359942	15	16	0.003881025	−1.76723	#	−2.38507	*	−2.37663	*
**BE**	10400	5	2080	0.511312	14	14	0.005512135	−0.94967	n.s.	−3.34368	*	−2.82884	*
**CR**	9000	4	2250	0.443434	11	11	0.004733045	−0.81801	n.s.	−2.47875	#	−2.14437	#
**NU**	5800	2	2900	0.246305	4	4	0.002559017	−0.08478	n.s.	1.061882	n.s.	0.784461	n.s.
**R1**	9600	6	1600	0.440603	13	13	0.004616837	−1.18725	n.s.	−1.51922	n.s.	−1.56665	n.s.
**R2**	9400	3	3133	0.232192	8	8	0.002460444	−1.31727	n.s.	−1.51372	n.s.	−1.60568	n.s.
**SA**	10000	4	2500	0.350204	7	7	0.003049033	−0.65838	n.s.	−1.09429	n.s.	−1.05868	n.s.
**SK**	6400	4	1600	0.421371	6	6	0.002691663	−0.85516	n.s.	0.422946	n.s.	0.041576	n.s.
**TL**	5400	6	900	0.495726	10	10	0.004366004	−1.14214	n.s.	−1.20381	n.s.	−1.27963	n.s.
**Scottish Mean**	**7917**	**4.6**	**2181.7**	**0.39**	**8.9**	**9**	**0.003600156**	**−0.90**		**−1.11**		**−1.14**	
**JA**	1000	3	333	0.7	8	8	0.011428571	1.027529	n.s.	1.027529	n.s.	1.027529	n.s.
**JB**	4200	2	2100	0.495238	6	6	0.007717996	2.455447	*	1.244184	n.s.	1.687244	**
**JC**	1800	1	1800	0	0	0	0	n.a.	n.a.	n.a.	n.a.	n.a.	n.a.
**JD**	5000	4	1250	0.59	6	6	0.004103896	−0.01694	n.s.	0.48652	n.s.	0.364349	n.s.
**JE**	7200	4	1800	0.620635	7	7	0.004638219	0.163899	n.s.	−0.19172	n.s.	−0.08969	n.s.
**JF**	5800	2	2900	0.46798	4	4	0.004862133	2.171078	*	1.061882	n.s.	1.490475	#
**JG**	2200	2	1100	0.436364	4	4	0.004533648	1.018279	n.s.	1.214656	n.s.	1.197402	n.s.
**JH**	7000	4	1750	0.448739	10	10	0.004801921	−0.73124	n.s.	−1.40457	n.s.	−1.30486	n.s.
**JI**	5600	4	1400	0.767196	6	6	0.005181062	0.852825	n.s.	1.2198	n.s.	1.19787	n.s.
**JJ**	4400	2	2200	0.311688	4	4	0.00323832	0.382533	n.s.	1.095481	n.s.	0.95141	n.s.
**JK**	5600	3	1867	0.420635	4	4	0.003689961	0.99969	n.s.	1.06593	n.s.	1.125182	n.s.
**JL**	6000	2	3000	0.331034	4	4	0.003439319	0.799666	n.s.	1.058022	n.s.	1.05968	n.s.
**JM**	6600	3	2200	0.363636	5	5	0.003886265	0.571243	n.s.	1.134768	n.s.	1.048538	n.s.
**JN**	5600	3	1867	0.603175	5	5	0.004535147	0.996995	n.s.	1.150643	n.s.	1.19031	n.s.
**JO**	5400	3	1800	0.646724	5	5	0.004454804	0.903574	n.s.	1.154262	n.s.	1.161935	n.s.
**JP**	4400	2	2200	0.415584	1	1	0.00107944	0.895275	n.s.	0.635044	n.s.	0.743867	n.s.
**JQ**	3200	2	1600	0.325	5	5	0.004220779	0.258449	n.s.	1.214388	n.s.	1.001992	n.s.
**JR**	4000	2	2000	0.189474	4	4	0.001968558	−0.94895	n.s.	1.10821	n.s.	0.569741	n.s.
**JS**	5000	4	1250	0.68	9	9	0.005766234	−0.22256	n.s.	−0.83848	n.s.	−0.70441	n.s.
**English Mean**	**4737**	**2.7**	**1811**	**0.46**	**5.1**	**5.1**	**0.004397172**	**0.64**		**0.75**		**0.76**	

Neutrality tests further differentiated the regions, with Scotland showing a negative and England positive TajimaD (−0.90 vs. 0.64), FuLiD* (−1.11 vs. 0.75), and FuLiF* (−1.14 vs. 0.76). The negative values in Scotland suggest an excess of low-frequency polymorphisms, indicating population expansion or purifying selection, and the positive values in England suggest balancing selection or a decrease in population size. While these variations were not statistically significant on most individual farms, the regional averages revealed pronounced and highly significant differences, with a p-value < 0.001 for all three tests.

### Comparison with publicly available *H. contortus* and *T. circumcincta* sequence data from different countries

**Fig 3:**
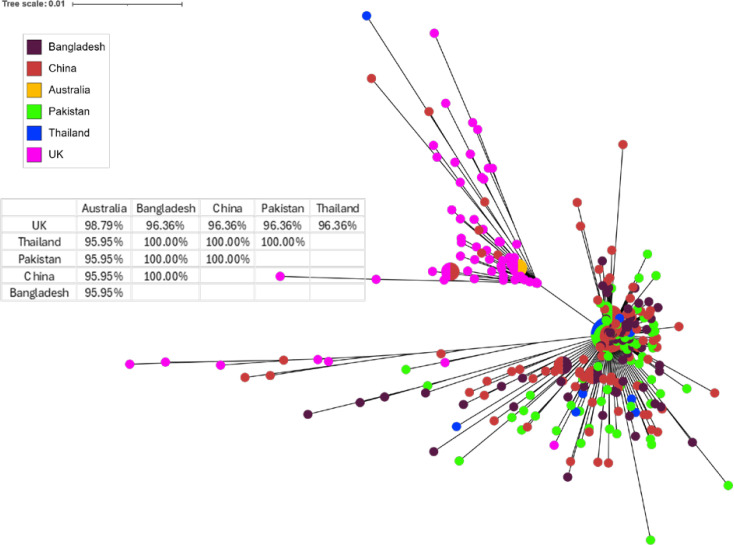
H. contortus mt-ND4 sequences from different countries. The phylogenetic tree illustrates the relationships among these ASVs found in this study and the matching sequences obtained from NCBI Genbank, originating in Bangladesh, China, Australia, Pakistan and Thailand. The UK samples represent the ones collected during this study. Each node represents a unique ASV, while the branches indicate the genetic distances between them. The colours represent different countries, while the node size represents the number of countries in which a sequence was found. The table shows the percentage similarity between sequences from different countries based on consensus sequences from each country.

**Fig 4:**
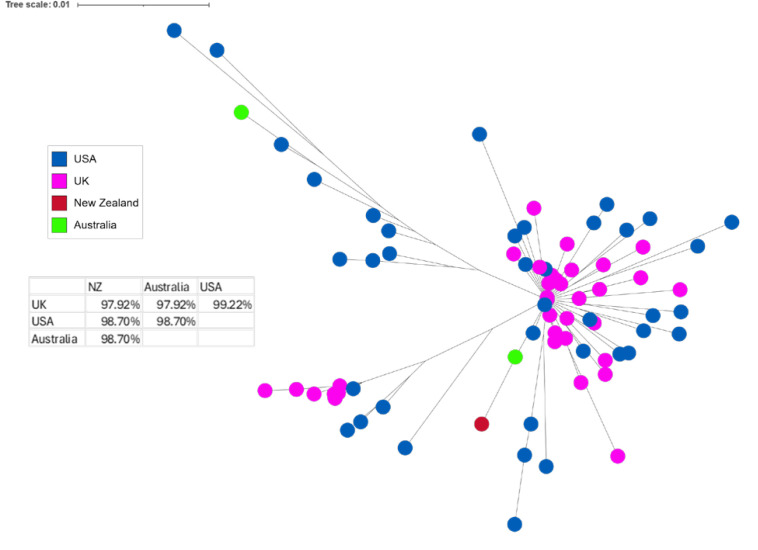
T. circumcincta mt-ND4 sequences from different countries. The phylogenetic tree illustrates the relationships among these ASVs found in this study and the matching sequences obtained from NCBI Genbank, originating in the USA, New Zealand and Australia. The UK samples represent the ones collected during this study. Each node represents a unique ASV, while the branches indicate the genetic distances between them. The colours represent different countries, while the node size represents the number of countries in which a sequence was found. The table shows the percentage similarity between sequences from different countries based on consensus sequences from each country.

Figs 3 and 4 show the phylogenetic trees for all available Genbank sequences for *H. contortus* and *T. circumcincta,* respectively, including the UK field samples collected during this study. For *H. contortus*, the available Genbank sequences that completely aligned with the targeted region mainly originated from Asian countries (Pakistan, Bangladesh, China and Thailand), except a single sequence from Australia. Despite some mixing, the tree shows two clear clusters: one based on the sequences from Asia, and the other one from the UK and Australia. The table in Fig 3 shows the percentage similarity between the countries based on the mutations present, and it shows the sequences originating from the Asian countries to have 100% similarity in comparison to only 95.95% similarity with the current UK samples.

For *T. circumcincta*, the Genbank sequences originated from New Zealand, Australia and the USA. The phylogenetic tree shows no clear differences based on the country of origin (Fig 4). The table shows that the UK samples were closer to the sequences originating from the USA (99.22% similarity) than any other countries, although this might be skewed by the low numbers of sequences available from those other countries.

## Discussion

The study describes the development of Illumina metabarcoding-adapted mt-ND4 primers for *H. contortus* and *T. circumcincta*, arguably the two most important GIN species infecting small ruminants in warm and temperate climates, respectively. These adapter primers offer an efficient way to study the genetic diversity patterns of GIN species with the possibility of examining hundreds of populations in a single run. Despite a somewhat limited sample set and challenges in field application, the study yielded insightful results regarding the genetic diversity patterns of *H. contortus* and *T. circumcincta* populations in the UK, with significant implications for understanding parasite dynamics and guiding control strategies.

The challenges faced in applying the mitochondrial multiplex PCR in field settings underscore the complexities of translating lab-validated methods to practical applications. The study reflects on possible improvements, noting that the DNA extraction method employed produced a crude extract [29] that might not have been entirely suitable for sensitive multiplex PCR applications due to potential PCR inhibitors [44–46]. Future studies could benefit from employing more refined DNA extraction methods to improve DNA quality and integrity, thereby enhancing the consistency of results across different samples [47].

The high genetic diversity observed in *H. contortus*, with 60 unique ASVs identified across UK farms, underscores the parasite’s evolutionary adaptability and genetic variability. *H. contortus* is recognised for its high reproductive output and extensive gene flow, both of which contribute to maintaining significant genetic diversity [48]. Additionally, its ability to infect a wide range of hosts (as explored in a related study [43]) further enhances its genetic variability, as different host species exert unique selective pressures on the parasite population [49].

The homogenisation of ASVs across farms in both English and Scottish regions likely reflects the frequent inter-farm movement of livestock, a common practice in the UK agricultural system [42,50]. Phylogenetic analysis suggests a common origin for all the *H. contortus* populations studied. However, the greater genetic differences observed in Scottish compared to English farms suggest localised selective pressures shaping population structure; likely driven by environmental factors reducing its prevalence as shown in the ‘nemabiome’ results (S2 Fig) but also potentially differing management practices [51].

The high genetic diversity in *H. contortus* populations has implications for developing anthelmintic resistance. Parasites with greater genetic variability are more likely to harbour alleles conferring resistance to treatments, providing a reservoir for selection under drug pressure [48]. Even though anthelmintic resistance is not extensively reported from *H. contortus* in the UK [37], the high genetic diversity of these populations equips them with the ability to adapt rapidly to changing selective pressures, such as those imposed by drug treatments. This adaptability represents a significant challenge in managing gastrointestinal nematodes and underscores the need for vigilant monitoring through molecular tools to detect resistance emergence at its earliest stages.

Most of the *T. circumcincta* and *H. contortus* populations displayed dominance by a small number of variants (ASVs). This was particularly noticeable in *T. circumcincta,* where the three most common ASVs made up over 85% of the total sequencing reads. Interestingly, *T. circumcincta* also demonstrated overall lower genetic diversity compared to *H. contortus*, with only 35 ASVs identified. This seems counterintuitive, given that genetic diversity often correlates with species survival and *T. circumcincta* is the more prevalent species. However, the unique epidemiological and pathogenic profiles of GINs make direct comparisons between species particularly challenging [5–9,52,53]. Hence, it is important to consider species-specific factors when interpreting genetic diversity and its implications for parasite management.

One possible explanation for the low genetic diversity in *T. circumcincta* is that its survival and persistence may rely on a limited number of well-established genetic variants. If these variants are already well-suited to prevailing conditions, further diversification may not be necessary, especially in the absence of strong selective pressures. This aligns with the species’ well-documented high levels of resistance to all major anthelmintics in the study area [54], suggesting that the existing genotypes are sufficient for maintaining populations under current management and environmental conditions. This context highlights the potential value of using these genetic markers with pre-treatment and post-treatment samples, such as from a faecal egg count reduction test (FECRT). Such an approach could provide insights into how treatments might influence the genetic diversity patterns of this parasite, offering a clearer understanding of its adaptability and potential resistance mechanisms.

The phylogenetic analysis suggested two distinct origins for *T. circumcincta* shared by populations in both regions. This could also lead to lower overall genetic diversity if there are mating barriers between distinct strains or subpopulations, similar to what has been reported in experimental genetic crossing studies for *H. contortus* strains [55]. Neutrality tests provide valuable insights into the evolutionary forces acting on populations by comparing observed genetic variation to expectations under a neutral model. For *T. circumcincta*, neutrality tests reveal contrasting evolutionary pressures between farms from both regions. In Scottish farms, the results suggest purifying selection or population expansion, likely resulting from favourable environmental conditions. Purifying selection removes deleterious alleles, promoting genetic stability and local adaptation. This selective process may also explain the lower but stable genetic diversity observed in *T. circumcincta*. In England, neutrality tests indicate balancing selection, or population contraction, potentially linked to inter-species competition [56] associated with the higher prevalence of *H. contortus*. Since *T. circumcincta* appears resistant to all major anthelmintics [54], this interspecies competition might be the only major bottleneck for its prevalence in the region.

Conversely, *H. contortus* showed indications of population contraction or balancing selection across populations from both regions. This might reflect the challenges this species faces, including susceptibility to anthelmintics, unfavourable climatic conditions and competition from other GINs. Such bottlenecks often decrease genetic diversity; however, in a species characterised by high reproductive rates and extensive gene flow [48], the effects of these contractions may be mitigated, allowing for the retention of significant genetic variability.

The low yet persistent prevalence of *H. contortus* on certain Scottish farms hints at unique local micro-environments, or management factors that might facilitate its survival on those individual farms while the overall expansion is still being kept in check by unfavourable large scale environmental conditions. The role of a potential reservoir or maintenance host in sustaining *H. contortus* populations, particularly on multi-host communities such as Farm A, is explored in further studies [43,57].

The comparison with previously available *H. contortus* sequences from GenBank reveals a clear genetic distinction between isolates from Asian countries and those from the UK and Australia. This separation likely reflects historical divergence and region-specific evolutionary pressures shaping the genetic structure of *H. contortus* populations [51,58]. In contrast, *T. circumcincta* does not exhibit significant geographic clustering, possibly due to the smaller dataset available for this species. These findings highlight the strength of Illumina-adapted mitochondrial markers, which, through high-throughput sequencing, enable large-scale genetic assessments across geographically diverse populations. By generating extensive sequence data from multiple regions, this approach allows for a more comprehensive understanding of genetic diversity, population connectivity, and the identification of distinct variants within important GIN species. Such insights are crucial for informing localised sustainable parasite management strategies.

Overall, these results underscore the complexity of GIN population dynamics and emphasise the importance of tailored management practices that account for local ecological conditions and the interplay of different GIN species. The study provides valuable insights into the genetic diversity and evolutionary dynamics of *H. contortus* and *T. circumcincta*; although the cross-sectional sampling design restricts the ability to infer temporal changes in genetic diversity and population dynamics. Additionally, while effective for detecting variation, mitochondrial markers do not capture the full scope of genetic diversity. Hence, these findings should be integrated with comprehensive genomic studies, such as whole genome sequencing or genome-wide locus sequence typing, before drawing definitive and actionable conclusions. Chromosomal-level genome assemblies are available for both *H. contortus* [59] and *T. circumcincta* [60], which can help with these types of studies. For instance, investigating the genetic differences between the two identified subpopulations of *T. circumcincta* could shed light on their potential impact on pathogenic traits, including resistance development.

Nevertheless, this study establishes foundational tools and lays a foundation for future research to explore the intricate ecological, genetic, and environmental factors driving GIN distribution and diversity across the UK and elsewhere. Such understanding is critical for developing sustainable, effective control strategies responsive to the evolving challenges of climate change and anthelmintic resistance.

## Supporting information

S1 Fig Flow chart of mt-ND multiplex development. The flowchart depicts the iterative process involved in the development of mt-ND multiplex.
(DOCX)

S2 Fig Relative abundance of different GIN species on individual farms. The bar chart shows the corrected read proportions of different GIN species on individual sheep farms in England and Scotland. Each bar is colour-coded to differentiate the species and includes mean faecal egg count (FEC) values at the top and farm codes at the bottom.
(DOCX)

S3 Fig Multiplex validation with known pools of *H. contortus* and *T. circumcincta* larvae. The table shows the number of L_3_ from each species present in different samples. The top bands show the presence of *T. circumcincta* (~385 base pairs) and the bottom bands represent *H. contortus* (~247 base pairs).
(DOCX)

S4 Fig Expected and obtained proportional reads of H. contortus and T. circumcincta. The top charts show the obtained proportional reads for each lab replicate described in the methods section. The bottom left chart shows the expected proportions based on the number of larvae in each sample, while the right chart shows the mean obtained reads in those samples. Sample 12 is not shown because it was empty in all replicates.
(DOCX)

S5 Fig The abundance and relationship of T. circumcincta and H. contortus ASVs. The distribution of reads attributed to each T. circumcincta (top) and H. contortus (bottom) ASV across samples, alongside maximum likelihood (ML) trees that delineate the relationships among the ASVs. The representation through stacked area charts demonstrates the consistency in the proportions of individual ASVs across different samples, regardless of the total read counts.
(DOCX)

S6 Fig Replicates of H. contortus and T. circumcincta laboratory strains. The bar charts present proportional reads for each H. contortus (top) and T. circumcincta (bottom) ASV. The legends show the 51 and 47 ASVs represented, respectively. The strain names are on the top along with one set of replicates of an unknown strain for T. circumcincta.
(DOCX)

S1 Table Mitochondrial ND4 Sequences used for primers design and as the reference library. The table lists the accession numbers of sequences used for the primer design of each species, along with the ones in the reference libraries that have been updated since then with the availability of more reference sequences.
(DOCX)

S2 Table The mitochondrial primers developed for *H. contortus* and *T. circumcincta.* The table lists forward and reverse primers developed for both species alongside the adapters and other modifications according to the Illumina sequencing requirements. There were eight primers for each species, making total of 16 that were combined for the multiplex.
(DOCX)
