# Supplementary material for: Development and field application of metabarcoding-adapted mt-ND4 markers shows substantial gene flow and varying local pressures on Haemonchus contortus and Teladorsagia circumcincta populations in the UK
Source: PLoS One. 2025 Jul 2;20(7):e0327254. doi: 10.1371/journal.pone.0327254 (PMC12221061; doi:10.1371/journal.pone.0327254)
Supplement: S1 Fig — The flowchart depicts the iterative process involved in the development of mt-ND multiplex. (DOCX) [file pone.0327254.s001.docx]

#
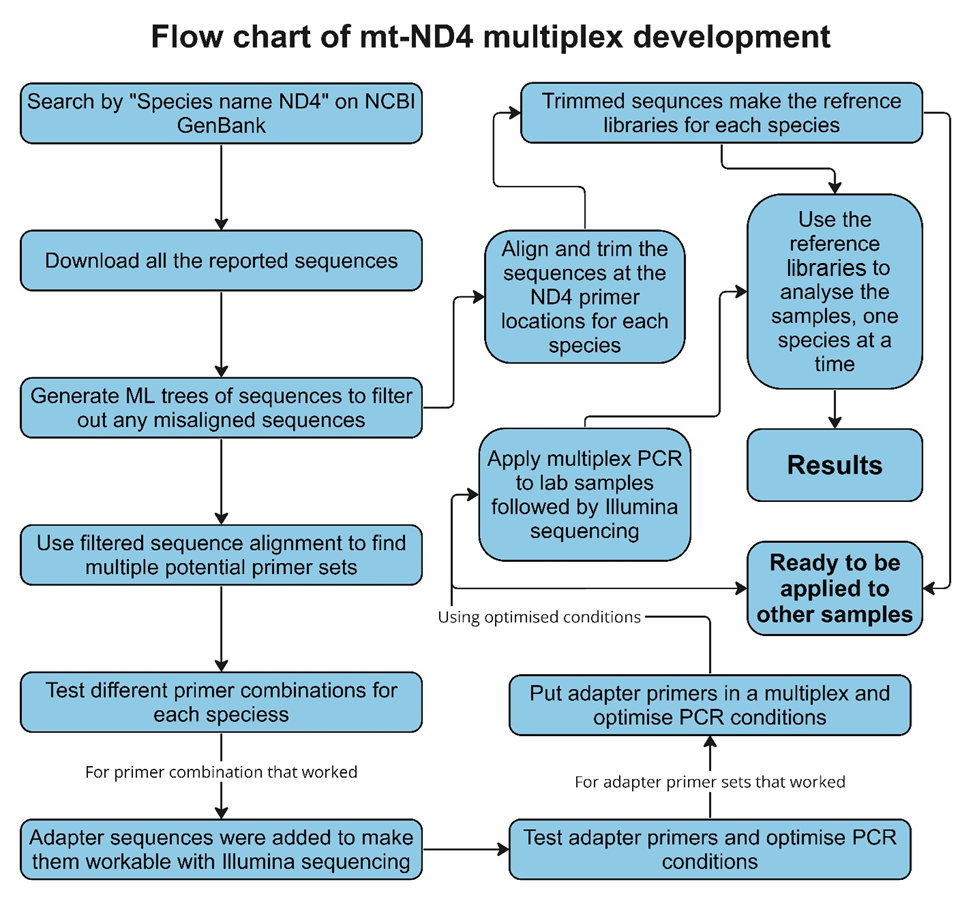


**Supplementary Figure 1:** **Flow chart of mt-ND multiplex development**

The flowchart depicts the iterative process involved in the development of mt-ND multiplex.
