# Supplementary material for: Development and field application of metabarcoding-adapted mt-ND4 markers shows substantial gene flow and varying local pressures on Haemonchus contortus and Teladorsagia circumcincta populations in the UK
Source: PLoS One. 2025 Jul 2;20(7):e0327254. doi: 10.1371/journal.pone.0327254 (PMC12221061; doi:10.1371/journal.pone.0327254)
Supplement: S2 Fig — The bar chart shows the corrected read proportions of different GIN species on individual sheep farms in England and Scotland. Each bar is colour-coded to differentiate the species and includes mean faecal egg count (FEC) values at the top and farm codes at the bottom. (DOCX) [file pone.0327254.s002.docx]

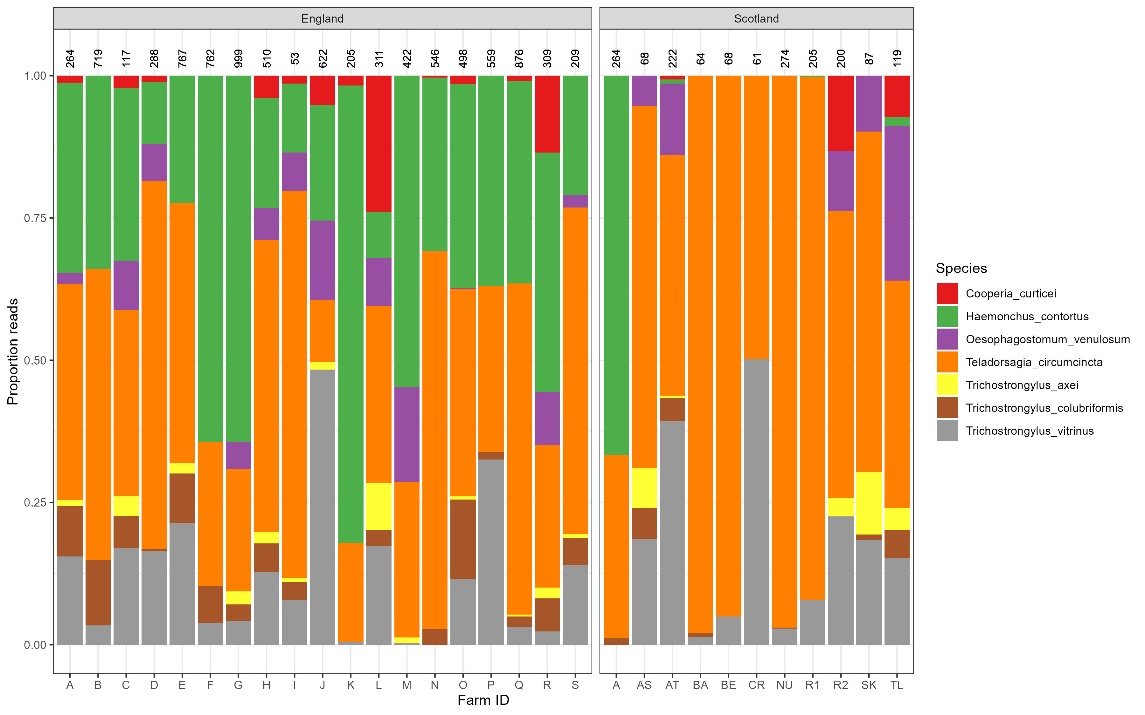


**Supplementary Figure 2:** **Relative abundance of different GIN species on individual farms**

The bar chart shows the corrected read proportions of different GIN species on individual sheep farms in England and Scotland. Each bar is colour-coded to differentiate the species and includes mean faecal egg count (FEC) values at the top and farm codes at the bottom.
