# Supplementary material for: Development and field application of metabarcoding-adapted mt-ND4 markers shows substantial gene flow and varying local pressures on Haemonchus contortus and Teladorsagia circumcincta populations in the UK
Source: PLoS One. 2025 Jul 2;20(7):e0327254. doi: 10.1371/journal.pone.0327254 (PMC12221061; doi:10.1371/journal.pone.0327254)
Supplement: S3 Fig — The table shows the number of L3 from each species present in different samples. The top bands show the presence of T. circumcincta (~385 base pairs) and the bottom bands represent H. contortus (~247 base pairs). (DOCX) [file pone.0327254.s003.docx]

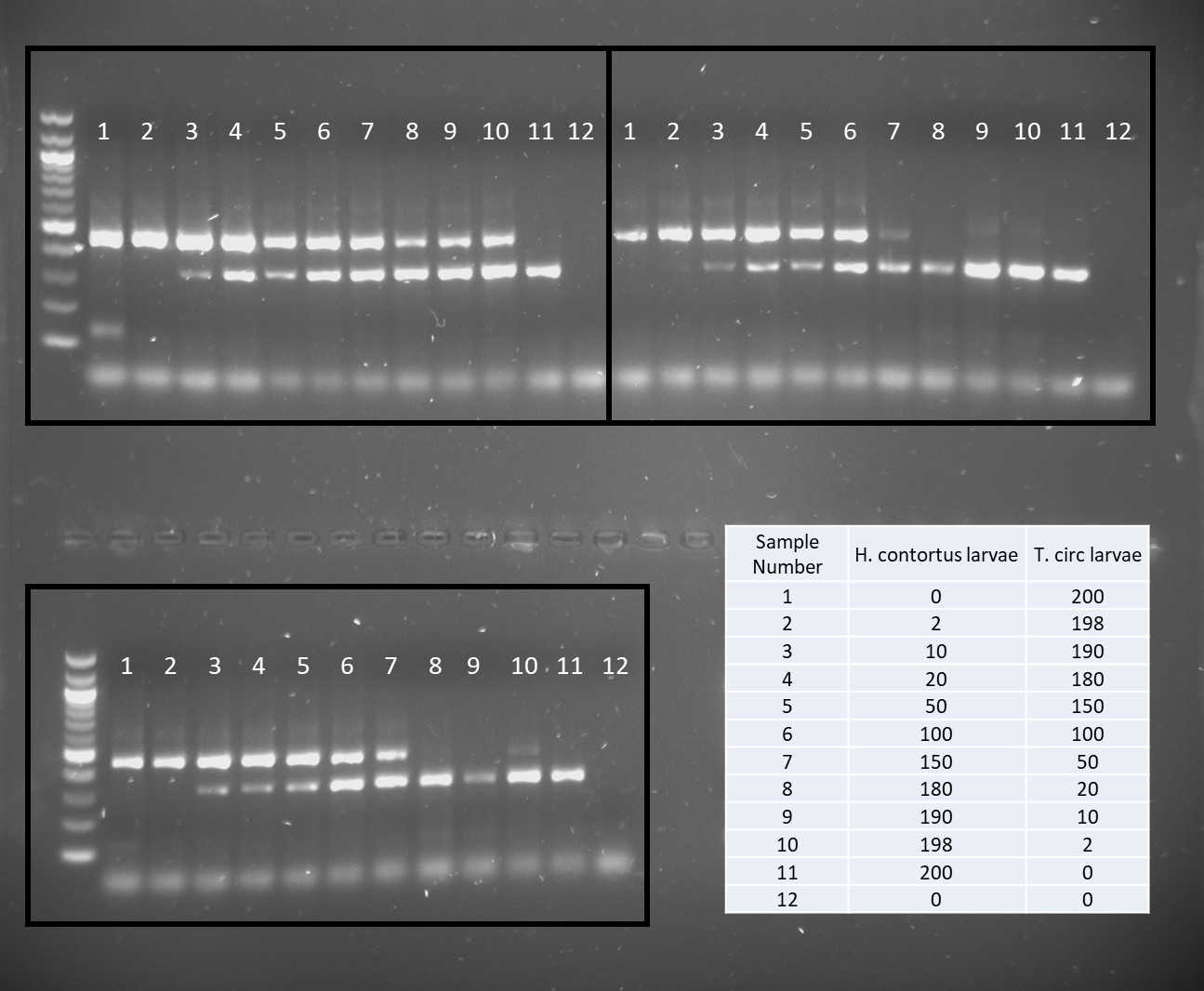


**Supplementary Figure 3:** **Multiplex validation with known pools of *H. contortus* and *T. circumcincta* larvae**

The table shows the number of L_3_ from each species present in different samples. The top bands show the presence of *T. circumcincta* (~385 base pairs) and the bottom bands represent *H. contortus* (~247 base pairs).
