# Supplementary material for: Development and field application of metabarcoding-adapted mt-ND4 markers shows substantial gene flow and varying local pressures on Haemonchus contortus and Teladorsagia circumcincta populations in the UK
Source: PLoS One. 2025 Jul 2;20(7):e0327254. doi: 10.1371/journal.pone.0327254 (PMC12221061; doi:10.1371/journal.pone.0327254)
Supplement: S4 Fig — The top charts show the obtained proportional reads for each lab replicate described in the methods section. The bottom left chart shows the expected proportions based on the number of larvae in each sample, while the right chart shows the mean obtained reads in those samples. Sample 12 is not shown because it was empty in all replicates. (DOCX) [file pone.0327254.s004.docx]

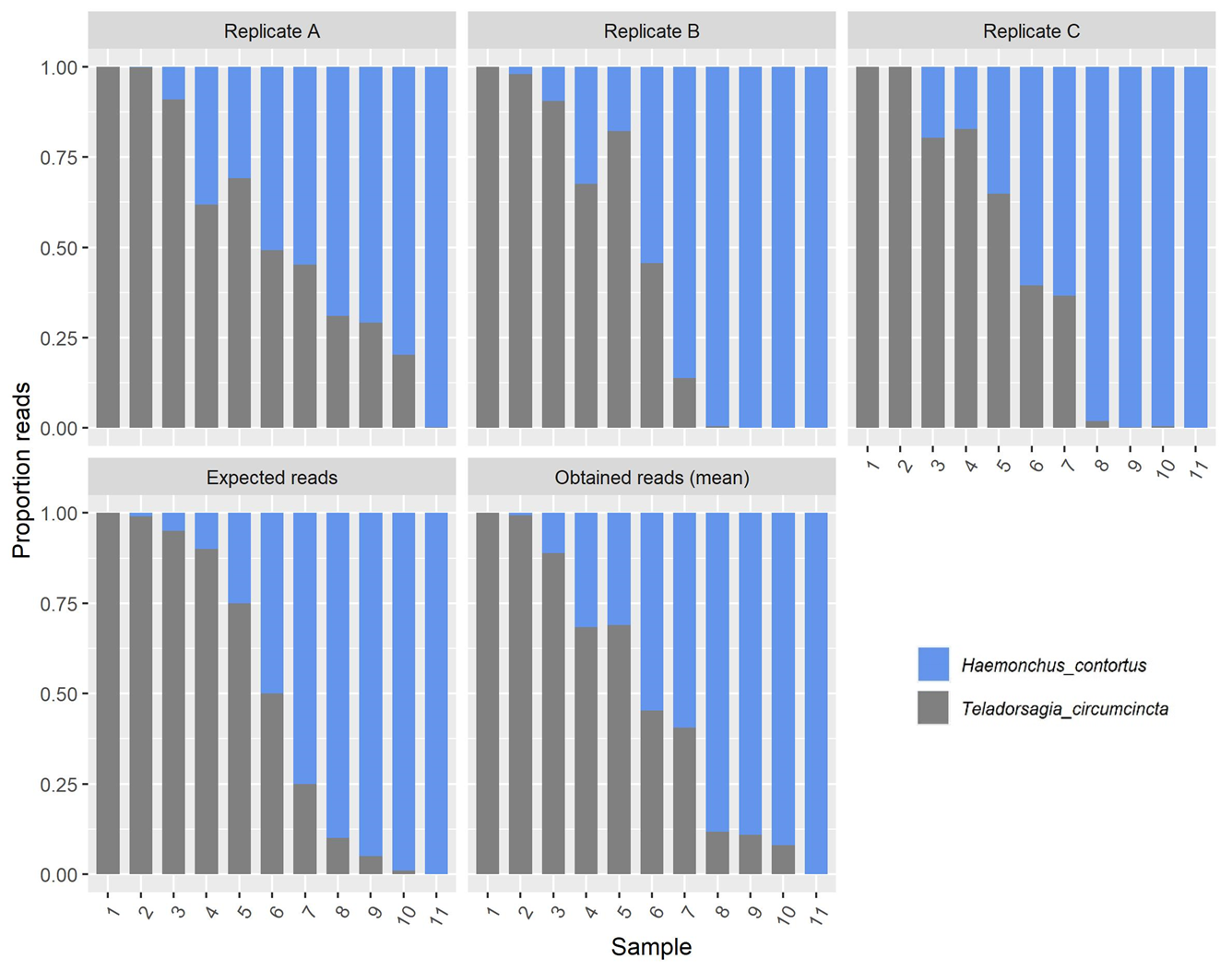


**Supplementary Figure 4:** **Expected and obtained proportional reads of H. contortus and T. circumcincta**

The top charts show the obtained proportional reads for each lab replicate described in section 2.4. The bottom left chart shows the expected proportions based on the number of larvae in each sample, while the right chart shows the mean obtained reads in those samples. Sample 12 is not shown because it was empty in all replicates.
