# Supplementary material for: Development and field application of metabarcoding-adapted mt-ND4 markers shows substantial gene flow and varying local pressures on Haemonchus contortus and Teladorsagia circumcincta populations in the UK
Source: PLoS One. 2025 Jul 2;20(7):e0327254. doi: 10.1371/journal.pone.0327254 (PMC12221061; doi:10.1371/journal.pone.0327254)
Supplement: S5 Fig — The distribution of reads attributed to each T. circumcincta (top) and H. contortus (bottom) ASV across samples, alongside maximum likelihood (ML) trees that delineate the relationships among the ASVs. The representation through stacked area charts demonstrates the consistency in the proportions of individual ASVs across different samples, regardless of the total read counts. (DOCX) [file pone.0327254.s005.docx]

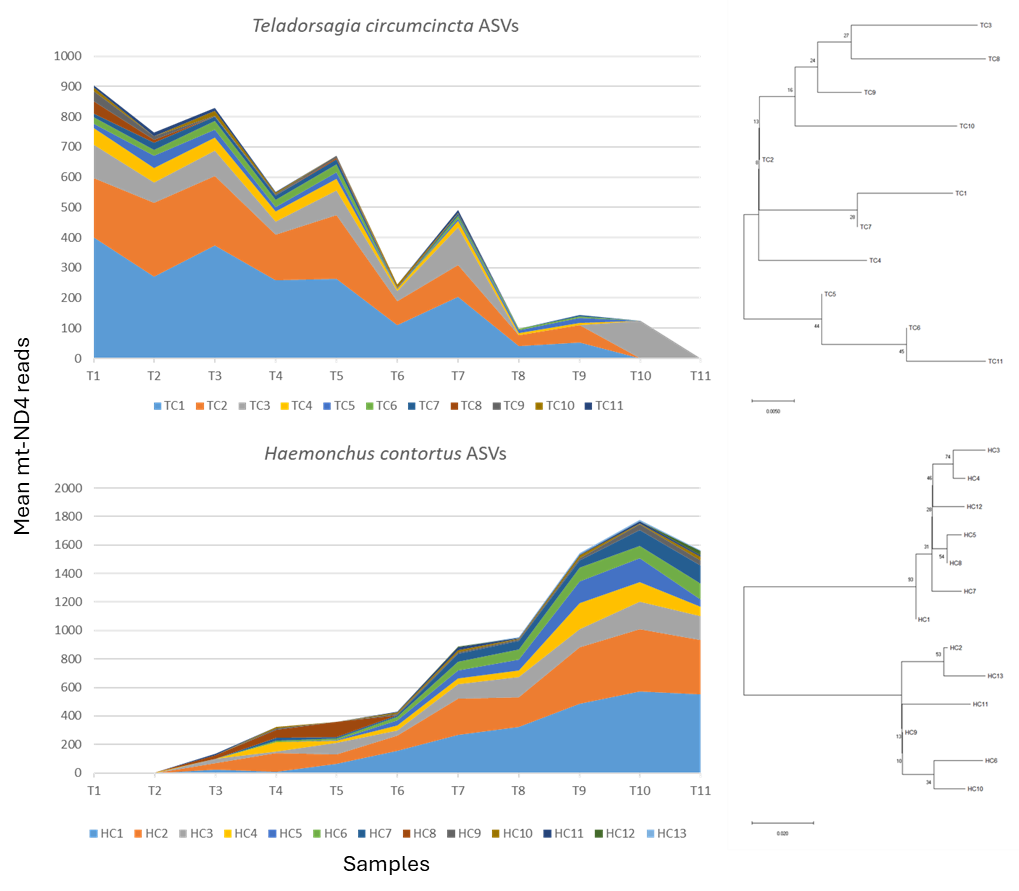


**Supplementary Figure 5: The abundance and relationship of T. circumcincta and H. contortus ASVs**

The distribution of reads attributed to each T. circumcincta (top) and H. contortus (bottom) ASV across samples, alongside maximum likelihood (ML) trees that delineate the relationships among the ASVs. The representation through stacked area charts demonstrates the consistency in the proportions of individual ASVs across different samples, regardless of the total read counts.
