# Supplementary material for: Development and field application of metabarcoding-adapted mt-ND4 markers shows substantial gene flow and varying local pressures on Haemonchus contortus and Teladorsagia circumcincta populations in the UK
Source: PLoS One. 2025 Jul 2;20(7):e0327254. doi: 10.1371/journal.pone.0327254 (PMC12221061; doi:10.1371/journal.pone.0327254)
Supplement: S6 Fig — The bar charts present proportional reads for each H. contortus (top) and T. circumcincta (bottom) ASV. The legends show the 51 and 47 ASVs represented, respectively. The strain names are on the top along with one set of replicates of an unknown strain for T. circumcincta. (DOCX) [file pone.0327254.s006.docx]

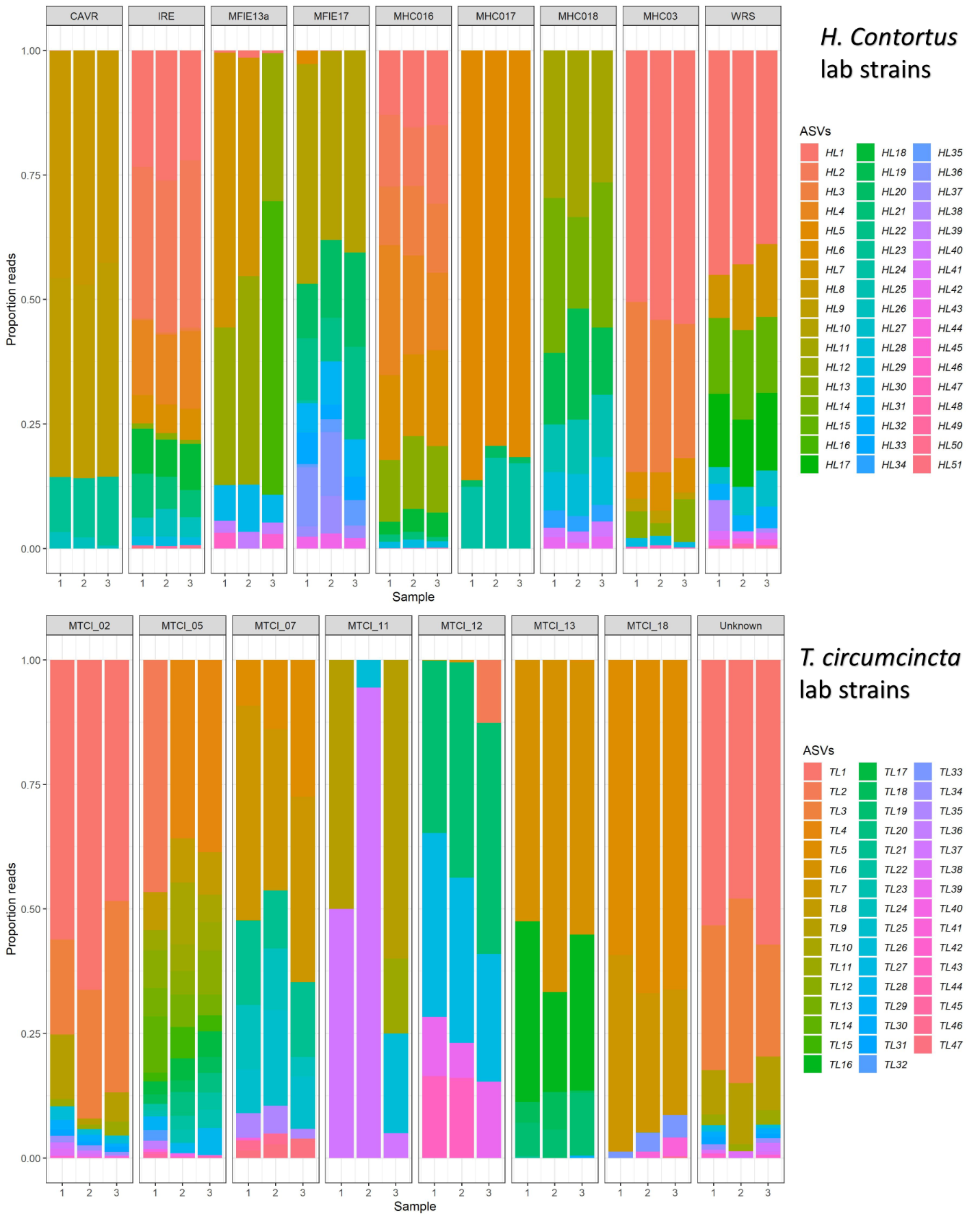


**Supplementary Figure 6: Replicates of H. contortus and T. circumcincta laboratory strains**

The bar charts present proportional reads for each H. contortus (top) and T. circumcincta (bottom) ASV. The legends show the 51 and 47 ASVs represented, respectively. The strain names are on the top along with one set of replicates of an unknown strain for T. circumcincta.
