# Supplementary material for: Development and field application of metabarcoding-adapted mt-ND4 markers shows substantial gene flow and varying local pressures on Haemonchus contortus and Teladorsagia circumcincta populations in the UK
Source: PLoS One. 2025 Jul 2;20(7):e0327254. doi: 10.1371/journal.pone.0327254 (PMC12221061; doi:10.1371/journal.pone.0327254)
Supplement: S1 Table — The table lists the accession numbers of sequences used for the primer design of each species, along with the ones in the reference libraries that have been updated since then with the availability of more reference sequences. (DOCX) [file pone.0327254.s007.docx]

**Supplementary Table 1: Mitochondrial ND4 Sequences used for primers design and as the reference library.** The table lists the accession numbers of sequences used for the primer design of each species, along with the ones in the reference libraries that have been updated since then with the availability of more reference sequences.

| Species | Sequences used for primer design | Current reference sequences |
| --- | --- | --- |
| *H. contortus* | EU346694.2, KJ724441.1, KJ724504.1, KJ724523.1, KY031959.1, KY041818.1, KY041827.1, KY041837.1, KY041839.1, KY041841.1, KY305810.1, MF380898.1, MF380901.1, MF380903.1, MF380914.1, NC_010383.2 | EU346694.2, KC429945.1, KC429946.1, KC429947.1, KC429948.1, KC429949.1, KC429950.1, KC429951.1, KC429952.1, KC429953.1, KC429954.1, KC429955.1, KC429956.1, KC429957.1, KC429958.1, KC429959.1, KC429960.1, KC429961.1, KC429962.1, KC429963.1, KC429964.1, KC429965.1, KC429966.1, KC429967.1, KC429968.1, KC429969.1, KC429970.1, KC429971.1, KC429972.1, KC429973.1, KC429974.1, KC429975.1, KC429976.1, KC429977.1, KC429978.1, KC429979.1, KC429980.1, KC429981.1, KC429982.1, KC429983.1, KC429984.1, KC429985.1, KC429986.1, KC429987.1, KC429988.1, KC429989.1, KC429990.1, KC429991.1, KC429992.1, KC429993.1, KC429994.1, KC429995.1, KC429996.1, KC429997.1, KC429998.1, KC429999.1, KC430000.1, KC430001.1, KC430002.1, KC430003.1, KC430004.1, KC430005.1, KC430006.1, KC430007.1, KC430008.1, KC430009.1, KC430010.1, KC430011.1, KC430012.1, KC430013.1, KC430014.1, KC430015.1, KC430016.1, KC430017.1, KC430018.1, KC430019.1, KC430020.1, KC430021.1, KC430022.1, KC430023.1, KC430024.1, KC430025.1, KC430026.1, KC430027.1, KC430028.1, KC430029.1, KC430030.1, KC430031.1, KC430032.1, KC430033.1, KC430034.1, KC430035.1, KC430036.1, KC430037.1, KC430038.1, KC430039.1, KC430040.1, KC430041.1, KC430042.1, KC430043.1, KC430044.1, KC430045.1, KC430046.1, KC430047.1, KC430048.1, KC430049.1, KC430050.1, KC430051.1, KC430052.1, KC430053.1, KC430054.1, KC430055.1, KC430056.1, KC430057.1, KC430058.1, KC430059.1, KC430060.1, KC430061.1, KC430062.1, KC430063.1, KC430064.1, KC430065.1, KC430066.1, KC430067.1, KC430068.1, KC430069.1, KC430070.1, KC430071.1, KC430072.1, KC430073.1, KC430074.1, KC430075.1, KC430076.1, KC430077.1, KC430078.1, KC430079.1, KC430080.1, KC430081.1, KC430082.1, KC430083.1, KC430084.1, KC430085.1, KJ724439.1, KJ724440.1, KJ724441.1, KJ724442.1, KJ724443.1, KJ724444.1, KJ724445.1, KJ724446.1, KJ724447.1, KJ724448.1, KJ724449.1, KJ724450.1, KJ724451.1, KJ724452.1, KJ724453.1, KJ724454.1, KJ724455.1, KJ724456.1, KJ724457.1, KJ724458.1, KJ724459.1, KJ724460.1, KJ724461.1, KJ724462.1, KJ724463.1, KJ724464.1, KJ724465.1, KJ724466.1, KJ724467.1, KJ724468.1, KJ724469.1, KJ724470.1, KJ724471.1, KJ724472.1, KJ724473.1, KJ724474.1, KJ724475.1, KJ724476.1, KJ724477.1, KJ724478.1, KJ724479.1, KJ724480.1, KJ724481.1, KJ724482.1, KJ724483.1, KJ724484.1, KJ724485.1, KJ724486.1, KJ724487.1, KJ724488.1, KJ724489.1, KJ724490.1, KJ724491.1, KJ724492.1, KJ724493.1, KJ724494.1, KJ724495.1, KJ724496.1, KJ724497.1, KJ724498.1, KJ724499.1, KJ724500.1, KJ724501.1, KJ724502.1, KJ724503.1, KJ724504.1, KJ724505.1, KJ724506.1, KJ724507.1, KJ724508.1, KJ724509.1, KJ724510.1, KJ724511.1, KJ724523.1, KY031955.1, KY031956.1, KY031957.1, KY031958.1, KY031959.1, KY031960.1, KY031961.1, KY031962.1, KY031963.1, KY031964.1, KY041808.1, KY041809.1, KY041810.1, KY041811.1, KY041812.1, KY041813.1, KY041814.1, KY041815.1, KY041816.1, KY041817.1, KY041818.1, KY041819.1, KY041820.1, KY041821.1, KY041822.1, KY041823.1, KY041824.1, KY041825.1, KY041826.1, KY041827.1, KY041828.1, KY041829.1, KY041830.1, KY041831.1, KY041832.1, KY041833.1, KY041834.1, KY041835.1, KY041836.1, KY041837.1, KY041838.1, KY041839.1, KY041840.1, KY041841.1, KY041842.1, KY305790.1, KY305791.1, KY305792.1, KY305793.1, KY305794.1, KY305795.1, KY305796.1, KY305797.1, KY305798.1, KY305799.1, KY305800.1, KY305801.1, KY305802.1, KY305803.1, KY305804.1, KY305805.1, KY305806.1, KY305807.1, KY305808.1, KY305809.1, KY305810.1, KY305811.1, KY305812.1, KY305813.1, KY305814.1, KY305815.1, KY305816.1, KY305817.1, KY305818.1, KY305819.1, KY305820.1, KY305821.1, KY305822.1, KY305823.1, KY305824.1, KY305825.1, KY305826.1, KY305827.1, KY305828.1, KY305829.1, KY305830.1, KY305831.1, KY305832.1, KY305833.1, KY305834.1, KY305835.1, KY305836.1, KY305837.1, KY305838.1, KY305839.1, KY305840.1, KY305841.1, KY305842.1, KY305843.1, KY305844.1, KY305845.1, KY305846.1, KY305847.1, KY305848.1, KY305849.1, KY305850.1, KY305851.1, KY305852.1, KY305853.1, KY305854.1, KY305855.1, KY305856.1, KY305857.1, KY305858.1, KY305859.1, KY305860.1, KY305861.1, KY305862.1, LC376827.1, LC376828.1, LC376829.1, LC376830.1, LC376831.1, LC376832.1, LC376833.1, LC376834.1, LC376835.1, LC376836.1, LC376837.1, LC376838.1, LC376839.1, LC376840.1, LC376841.1, LC376842.1, LC376843.1, LC376844.1, LC376845.1, LC376846.1, LC376847.1, LC376848.1, LC376849.1, MF380898.1, MF380899.1, MF380900.1, MF380901.1, MF380902.1, MF380903.1, MF380904.1, MF380905.1, MF380906.1, MF380907.1, MF380908.1, MF380909.1, MF380910.1, MF380911.1, MF380912.1, MF380913.1, MF380914.1, MF380915.1, MF380916.1, NC_010383.2 |
| *T. circumcincta* | AF070877.1, AF070881.1, AF070883.1, AF070887.1, AF070890.1, AF070894.1, AF070895.1, AF070896.1, AF070897.1, AF070898.1, AF070899.1, AF070900.1, AF070902.1, AF070904.1, AF070907.1, AF070908.1, AF070912.1, AF070914.1, AF070915.1, AF070916.1, AP017699.1, GQ888720.1, MN013406.1 | AF070877.1, AF070878.1, AF070879.1, AF070880.1, AF070881.1, AF070882.1, AF070883.1, AF070884.1, AF070885.1, AF070886.1, AF070887.1, AF070888.1, AF070889.1, AF070890.1, AF070891.1, AF070892.1, AF070893.1, AF070894.1, AF070895.1, AF070896.1, AF070897.1, AF070898.1, AF070899.1, AF070900.1, AF070901.1, AF070902.1, AF070903.1, AF070904.1, AF070905.1, AF070906.1, AF070907.1, AF070908.1, AF070909.1, AF070910.1, AF070911.1, AF070912.1, AF070913.1, AF070914.1, AF070915.1, AF070916.1, AP017699.1, GQ888720.1, MN013406.1, NC_013827.1 |
