# Supplementary material for: Development and field application of metabarcoding-adapted mt-ND4 markers shows substantial gene flow and varying local pressures on Haemonchus contortus and Teladorsagia circumcincta populations in the UK
Source: PLoS One. 2025 Jul 2;20(7):e0327254. doi: 10.1371/journal.pone.0327254 (PMC12221061; doi:10.1371/journal.pone.0327254)
Supplement: S2 Table — The table lists forward and reverse primers developed for both species alongside the adapters and other modifications according to the Illumina sequencing requirements. There were eight primers for each species, making total of 16 that were combined for the multiplex. (DOCX) [file pone.0327254.s008.docx]

**Supplementary Table 2: The mitochondrial primers developed for *H. contortus* and *T. circumcincta.*** The table lists forward and reverse primers developed for both species alongside the adapters and other modifications according to the Illumina sequencing requirements. There were eight primers for each species, making total of 16 that were combined for the multiplex.

| Species | Primer name | Type | Adapter | Modifications | Primer sequence |
| --- | --- | --- | --- | --- | --- |
| *H. contortus* | Hc5ND4 | Forward | TCGTCGGCAGCGTCAGATGTGTATAAGAGACAG | 0N | TTTGTGTATTTCAAAGTGATTC |
| *H. contortus* | Hc5ND4 | Forward | TCGTCGGCAGCGTCAGATGTGTATAAGAGACAG | 1N | TTTGTGTATTTCAAAGTGATTC |
| *H. contortus* | Hc5ND4 | Forward | TCGTCGGCAGCGTCAGATGTGTATAAGAGACAG | 2N | TTTGTGTATTTCAAAGTGATTC |
| *H. contortus* | Hc5ND4 | Forward | TCGTCGGCAGCGTCAGATGTGTATAAGAGACAG | 3N | TTTGTGTATTTCAAAGTGATTC |
| *H. contortus* | Hc5ND4 | Reverse | GTCTCGTGGGCTCGGAGATGTGTATAAGAGACAG | 0N | ATACTAAAAATTATACTAGAA |
| *H. contortus* | Hc5ND4 | Reverse | GTCTCGTGGGCTCGGAGATGTGTATAAGAGACAG | 1N | ATACTAAAAATTATACTAGAA |
| *H. contortus* | Hc5ND4 | Reverse | GTCTCGTGGGCTCGGAGATGTGTATAAGAGACAG | 2N | ATACTAAAAATTATACTAGAA |
| *H. contortus* | Hc5ND4 | Reverse | GTCTCGTGGGCTCGGAGATGTGTATAAGAGACAG | 3N | ATACTAAAAATTATACTAGAA |
| *T. circumcincta* | UN1ND | Forward | TCGTCGGCAGCGTCAGATGTGTATAAGAGACAG | 0N | TAACTATAAGAAGTAAAGTGGC |
| *T. circumcincta* | UN1ND | Forward | TCGTCGGCAGCGTCAGATGTGTATAAGAGACAG | 1N | TAACTATAAGAAGTAAAGTGGC |
| *T. circumcincta* | UN1ND | Forward | TCGTCGGCAGCGTCAGATGTGTATAAGAGACAG | 2N | TAACTATAAGAAGTAAAGTGGC |
| *T. circumcincta* | UN1ND | Forward | TCGTCGGCAGCGTCAGATGTGTATAAGAGACAG | 3N | TAACTATAAGAAGTAAAGTGGC |
| *T. circumcincta* | UN3ND | Reverse | GTCTCGTGGGCTCGGAGATGTGTATAAGAGACAG | 0N | CAAGTTACCCCTAAATTTCAATT |
| *T. circumcincta* | UN3ND | Reverse | GTCTCGTGGGCTCGGAGATGTGTATAAGAGACAG | 1N | CAAGTTACCCCTAAATTTCAATT |
| *T. circumcincta* | UN3ND | Reverse | GTCTCGTGGGCTCGGAGATGTGTATAAGAGACAG | 2N | CAAGTTACCCCTAAATTTCAATT |
| *T. circumcincta* | UN3ND | Reverse | GTCTCGTGGGCTCGGAGATGTGTATAAGAGACAG | 3N | CAAGTTACCCCTAAATTTCAATT |
